# Pain and Problem Behavior in Cats and Dogs

**DOI:** 10.3390/ani10020318

**Published:** 2020-02-18

**Authors:** Daniel S. Mills, Isabelle Demontigny-Bédard, Margaret Gruen, Mary P. Klinck, Kevin J. McPeake, Ana Maria Barcelos, Lynn Hewison, Himara Van Haevermaet, Sagi Denenberg, Hagar Hauser, Colleen Koch, Kelly Ballantyne, Colleen Wilson, Chirantana V Mathkari, Julia Pounder, Elena Garcia, Patrícia Darder, Jaume Fatjó, Emily Levine

**Affiliations:** 1 Animal Behavior, Cognition & Welfare Group, University of Lincoln, Lincoln LN6 7DL, UK; kmcpeake@lincoln.ac.uk (K.J.M.); abarcelos@lincoln.ac.uk (A.M.B.); lhewison@lincoln.ac.uk (L.H.); hVanHaevermaet@lincoln.ac.uk (H.V.H.); jpounder@lincoln.ac.uk (J.P.); 2 Behavior Medicine Department, Centre vétérinaire DMV, Montréal, QC H8T 3R2, Canada; isabelle.demontigny-bedard@umontreal.ca; 3 Department of Clinical Sciences, North Carolina State University College of Veterinary Medicine, Raleigh, NC 27606, USA; megruen@ncsu.edu; 4 Veterinary Behavior Consultant, Sainte-Anne-de-Bellevue, QC H9X0A6, Canada; maryklinck@hotmail.com; 5 Langford Veterinary Services, University of Bristol, Langford BS40 5DU, UK; sagidvm@gmail.com; 6 North Toronto Veterinary Behavior Specialty Clinic, Thornhill, ON L3T 2K9, Canada; 7 Department of Clinical Studies, School of Veterinary Medicine, University of Pennsylvania, 3900 Delancey Street, Philadelphia, PA 19104, USA; hhauser@vet.upenn.edu; 8 Animal Behavior Services, Lincoln Land Animal Clinic, Jacksonville, IL 62650, USA; lincolnlandac@yahoo.com; 9 Insight Animal Behavior Services, P.C., 815 W. Randolph St., Chicago, IL 60607, USA; drb@insightfulanimals.com; 10 1217 Wildfern Way, Greely, ON K4P-1R4, Canada; Colleenwilson@rogers.com; 11 Department of Animal and Avian Sciences, University of Maryland, College Park, Maryland, MD 20742, USA; mathkari@umd.edu; 12 Ars Veterinaria Hospital. Carrer dels Cavallers, 37, 08034 Barcelona, Spain; etovet.elena@gmail.com (E.G.); etologia.darder@gmail.com (P.D.); jaumefatjo@gmail.com (J.F.); 13 Animal Behavior Clinic of New Jersey, 240 Humphrey St, Englewood, NJ 07631, USA; dremilylevine@hotmail.com

**Keywords:** adjunctive behavior, aggression, attention seeking, compulsive behavior, house-soiling, pain, pica, stereotypy

## Abstract

**Simple Summary:**

The potential role of pain in problem behavior is widely acknowledged, but there seems to be a lack of reporting of this issue. It is difficult to present definitive evidence concerning the breadth of the problem given the individuality of problem behavior. In this commentary, we present evidence from our own caseloads to illustrate the scale and the nature of the issue with a view to increasing awareness of the problem by veterinarians, non-veterinary behaviorists, and owners. Among the referral caseloads of several of the authors, the prevalence in recent years ranges from 28–82%, and many of these conditions can be suspected from close observation of the patient. While the actual mechanism underpinning the association between pain and problem behavior may never be known in a given case, we suggest the relationship between the problem behavior and pain can be classified into one of four categories: the presenting complaint is a direct manifestation of pain; unidentified pain is underpinning secondary concerns within the initial behavior problem; there is an exacerbation of one or more signs of problem behavior as a result of pain; or adjunctive behavioral signs are associated with pain. We conclude that, in general, it is better for veterinarians to treat suspected pain first rather than consider its significance only when the animal does not respond to behavior therapy.

**Abstract:**

We argue that there is currently an under-reporting of the ways in which pain can be associated with problem behavior, which is seriously limiting the recognition of this welfare problem. A review of the caseloads of 100 recent dog cases of several authors indicates that a conservative estimate of around a third of referred cases involve some form of painful condition, and in some instances, the figure may be nearly 80%. The relationship is often complex but always logical. Musculoskeletal but also painful gastro-intestinal and dermatological conditions are commonly recognized as significant to the animal’s problem behavior. The potential importance of clinical abnormalities such as an unusual gait or unexplained behavioral signs should not be dismissed by clinicians in general practice, even when they are common within a given breed. In general, it is argued that clinicians should err on the side of caution when there is a suspicion that a patient could be in pain by carefully evaluating the patient’s response to trial analgesia, even if a specific physical lesion has not been identified.

## 1. Introduction

Associations between certain forms of common behavior problems in dogs (i.e., aggressive behavior, noise sensitivities) and chronic musculoskeletal pain have recently been described [1,2,3], and clearly there is a need to differentiate these pain-related conditions from ones that do not feature pain. However, pain-related effects may not manifest directly in a primary behavior complaint, but rather they may moderate a pre-existing behavioral condition or present unusual signs in association with a case. In the latter instance, it is important for clinicians to be sensitive to these potential markers of pain alongside more obvious ones. In this paper, we not only review current literature on the subject but also report on the outcomes of a workshop held at the 12th International Veterinary Behavior Meeting (IVBM) held in Washington DC in 2019 [4] alongside the experience of the authors in both primary and referral veterinary practice. We begin with a brief review of 100 recent dog behavior cases from several of the authors, which serves to highlight how common this issue is, since this issue has perhaps not received the recognition it deserves (Table 1). Historically, medical issues in relation to behavior problems appear to be becoming more frequent; the first report by Voith [5] indicated a 5% prevalence, with Mills [6] later reporting a 12% prevalence in dogs and 19% prevalence in cats. In 2013, an unpublished review of the case log of resident Karagiannis at the University of Lincoln returned a figure of 23% for dogs. This now appears to be at the lower end of the prevalence reported more recently across a wider geographic base by some of the authors of this article (Table 1).

The nature of the relationship between pain and problem behavior may be complex and heterogeneous but deserves consideration, and the aim of this paper is to primarily increase awareness of and interest in this topic. We acknowledge at the outset that some of these associations are simply observed correlations, and the exact relationship (if any) with pain needs further elucidation; however, amongst the authors, there is experience of such cases being confirmed diagnostically or the signs co-varying with analgesic management. Only by recognizing the potential role of pain can we start to build up the evidence base, and this paper hopes to raise awareness in the interests of protecting animal welfare. We would argue that we should adhere to the precautionary principle and seek to treat pain wherever we believe it could be involved. In order to bring together a range of observations that we believe deserve further consideration as potential indicators of discomfort, we consider the subject under the following four headings:

- Presenting complaint as a direct manifestation of pain;
- Unidentified pain underpinning secondary concerns within the initial behavior problem;
- Exacerbation of one or more signs of problem behavior as a result of pain;
- Adjunctive behavioral signs associated with pain.

## 2. Presenting Complaint as a Direct Manifestation of Pain

An animal in pain will naturally be more cautious and potentially anxious as a result. In this section, we consider specific behavioral problems that are caused by pain, while in later sections, we consider less direct relationships. The issue of the influence of pain on anxiety and problem behavior as a result is discussed in the third section dealing with the exacerbation of signs of pain, since, in our experience, this is the more common manifestation of this influence, but—as we illustrate in a case study in that section—some apparently anxious behavior problems may be directly related to pain.

### 2.1. Defensive Behavior

Within this category are a range of behavior problems, but perhaps most widely recognized are forms of aggressive behavior, since agonistic behavior serves to help avoid contact with humans or other animals. Barcelos and colleagues [2], in a qualitative review of cases of aggressive behavior in dogs with and without musculoskeletal pain, suggest a number of clinical features that might aid in (but are not diagnostically sufficient for) the recognition of pain involvement in these cases. Typically, these animals are often described as having a poor and changeable temperament, with terms such as the dog having a “Jekyll and Hyde” type of personality frequently being used. The aggressive behavior typically occurs when the dog is approached and often when the dog is lying down; further investigation of the background behavior of the dog also often reveals a more general reluctance to move. The authors also identified certain features of the bite that were typical of dogs with chronic musculoskeletal problems. The targets were often less specific, consisting of both familiar and unfamiliar individuals (whether they be dogs or humans), and the bites were often of variable severity and typically directed towards the limb extremities of the target (by contrast, dogs not in pain often delivered more severe bites to a wider diversity of body regions, including the face and the torso in addition to limbs). The bite incidents were also typically short and easy to interrupt. Taken together, these signs are strongly suggestive of the bites being a low level violent threat aimed at saving the animal from further interaction. It seems likely that lower level non-violent signals, such as head turns and hard stares, which are described as preludes to a bite in the “ladder of aggression” [7], have been ignored or not noticed prior to this and possibly may no longer be expressed as a result. This could make the behavior appear sudden and without warning. It has been reported that cases who were not aggressive before the onset of pain may appear more impulsive [1], while those cases that had shown aggression before the onset of the problem appear to be less impulsive but more intense or frequent in their aggressive displays. This is consistent with the predicted learning effects associated with differing levels of feedback from the consequences of their behavior over time. It is interesting to note that Barcelos et al. [2] also record that these problems often presented in younger dogs, and we speculate that this might be when they are learning which signals are most effective. Older dogs might learn to suppress these signs (but may still be in pain) or face elimination from the home in one way or another. The following is a typical case history.

- Case study: Aggression in a Dachshund associated with back pain

A 5.5-year-old, 7.9 kg male neutered miniature Dachshund mix was presented by referral for aggression towards owner. The dog had a 3–4 month history of lunging, baring his teeth, and snapping at the owner while resting near her on the bed or the couch, most often in the evening or immediately before bedtime. This would happen when the owner moved or shifted but did not necessarily touch the dog. Events were not entirely consistent and would happen 3–4 times/week. As part of the behavioral history evaluation, the owner also reported a recent onset of reluctance by the dog to walk when on lead. The referring veterinarian described their clinical examination as being unremarkable. There was mild to moderate anxiety in the hospital, and the referring veterinarian indicated that the patient was muzzled for examinations. Complete blood count and biochemistry were reported as being within normal limits, a thyroid profile, including TT4, TT3, free T4, and TSH, was also within normal limits. The dog did have a history of acute intermittent back pain ~4 years previously. During the consultation, the clinician observed intermittent trembling on the left rear limb alongside consistent off-loading of weight from the left to the right limb while standing. The dog also had a stilted hind limb gait bilaterally. All of these signs were interpreted as being indicative of discomfort. Accordingly, 4 weeks treatment with a non-steroidal anti-inflammatory drug (NSAID) (grapiprant) was recommended. A consultation with a veterinary rehabilitation specialist for further evaluation and physiotherapy was also arranged. In the interim, management and behavioral recommendations including giving the patient another area to rest, avoiding disturbing him when he was resting, and monitoring his body language closely for signs of threat and avoiding confrontation were recommended. The veterinary rehabilitation therapist suspected iliopsoas strain or intervertebral disc disease based on their physical examination, and additional diagnostics were not pursued at this point. Two months later, there had only been two incidents of aggression towards the owner in the 8–9 weeks since the initial consultation, and pain management protocol had been started. Both incidents happened when the dog was resting next to the owner and she shifted her weight or moved. Both owner and dog walker reported improvement in patient’s interest in walking on the lead without specific behavior modification being applied.

The published literature also describes case reports of specific ostensibly behavioral cases involving dogs presenting for aggression that were due to pain related to otitis [1,8], an arachnoid cyst [9], and diskospondylitis [10].

Specific reports of similar chronic pain related problem aggression in cats appear to be missing in the literature, but such responses are believed to occur in relation to both arthritis and dental disease [11,12,13,14] and typically manifest as poorer mood [13] and defensiveness in relation to interaction [13,15,16] but also increased fearfulness in some cases [13]. A retrospective cohort study of 137 declawed cats indicated that declawed cats were at increased risk (odds ratio of 2.66) of having back pain and nearly nine times more likely to show signs of aggression [17]. Central sensitization (sometimes called “wind-up pain”), in which there is increased sensitivity towards pain in body regions not initially affected by a painful lesion, is also believed to occur in the cat, resulting in aggression when touched in this non-painful region [18]; the absence of a lesion associated with the area may lead to the mistaken supposition that the cat’s problem is “behavioral” rather than pain-related. Evidence of central sensitization has been shown in cats with a lowering of thresholds for response during quantitative sensory testing [19,20]. The less commonly recognized condition of vomeronasalitis has also been associated with aggression in the cat: 5/20 cats with the condition showed intraspecific aggressive behavior and 8/20 showed human-related aggression [21]. The extent to which this might be pain related remains unknown but deserves consideration. In cats, gastro-intestinal pain associated with constipation secondary to congenital hypothyroidism may result in aggressive behavior, especially when petted [22].

- Case study: Aggression fearfulness and house-soiling problems in a cat associated with chronic pain

An 8-year-old male neutered domestic shorthaired cat was presented for intermittent urination outside of the litter box for approximately 4 years, which was now increasing. He would urinate on horizontal surfaces including: sleeping bags, in the sink, on dishes, the table top, and the automatic feeder. He shared the household with another cat, and they had always got along well; they would allogroom and rest together. However, more recently, there had been a lot of posturing and vocalization between the cats, although there had been no overt fights. The patient was very friendly to people and would greet guests when they would come into the house. Over the last 4 years, the patient had become increasingly more fearful of fireworks and “heavy” trucks passing by the house and would run downstairs. Multiple urinalyses had generally but not consistently revealed struvite crystals and white blood cells, with oxalate crystals occasionally evident. Several ultrasounds revealed thickening of the bladder wall. The patient had been treated with diet change(s) and antibiotics as warranted. Radiographs of the bladder were taken at the time of presentation for the behavior problem to ensure there were no uroliths. The radiographs revealed no uroliths, but arthritic changes in thoracolumbar spine were noted.

The cat was treated with over the counter joint supplements and gabapentin at 10.4mg/kg q12 hr. After 6 months with no incidents of aggression or elimination outside of the box, the gabapentin was reduced to 5.2 mg/kg. At the lower dose, signs of fear, elimination outside of the box, and aggression returned. The gabapentin dose was increased and maintained at 10.4 mg/kg q12 hr. The patient has since been without behavioral incidents for a period of 18 months.

### 2.2. Changes in Learning and Performance, Especially in Working Animals

Pain may result in overt signs such as lameness; however, even in the absence of overt pain-specific signs, pain has the potential to impact the learning and the performance of dogs. Apparently, poor learning in obedience classes, for example, not learning to sit properly, may arise as a result of the pain associated with placing dysplastic hips into that posture, and this can occur even in puppy classes. Performance problems related to pain can also be manifested only during specific movements (e.g., one-sided difficulty where there is a localized, unilateral source of pain) or can have a more general effect on performance (e.g., slowness or reluctance). This might be noted at the start of new training (e.g., in the case of painful developmental conditions such as hip dysplasia), or performance changes could arise in a trained dog over time (e.g., as a result of injury or the development of degenerative joint disease). Studies conducted in police and military working dogs support the impact of pain on performance. In a study of causes of loss or retirement of New Zealand police German Shepherd dogs, “inability to cope with the physical demands of the job” was often associated with degenerative musculoskeletal or spinal disease [23]. Similarly, a study of military working dogs found the latter disorders to be common medical reasons for discharge [24]. Recently, Bowen et al. [25] also found joint laxity to be associated with excitability in assistance dogs. Behavioral signs associated with lumbosacral stenosis in military working dogs included lethargy, being slow to rise, reluctance to search high, and reluctance to jump [26]. Another study of lumbosacral stenosis in military working dogs found the following behavior problems to be more likely in dogs with multi-level stenosis: “unwilling or reluctant to jump up onto objects/into vehicles” (38%), and “unwilling or reluctant to sit”, “handler-reported unusual behaviors”, as well as “increase in anxiety”, “sudden onset of aggressive behaviors”, “self-mutilation in the lower back region, tail, or hind legs”, each in 25% of cases [27]. Some of the better quality canine pain and health-related quality of life scales contain valuable items that relate to performance (work or sport), and these can be useful during the screening of dogs. Items related to activity during exercise include “activity level at exercise”, “keenness to exercise”, “frequency of rest during exercise” [Liverpool Osteoarthritis in Dogs (LOAD) scale, [28]], “willingness to trot/gallop/walk/jump”, and “ease in rising/lying down” (Helsinki Chronic Pain Index, [29]). In the GUVQuest, a health-related quality of life scale for dogs, items such as “athletic”, “consistent”, “energetic”, and “fit” distinguished between dogs with and without painful chronic disease [30]. It therefore seems prudent to evaluate for a cause of pain when a dog’s performance is altered, be it in police or military work, assistance, sport, hobby, or other work.

- Case study: Reduced performance in freestyle associated with chronic pain

The associated video (Supplementary Materials S1) is of a 13-year-old female spayed Weimaraner performing a canine freestyle routine. The dog had previously participated enthusiastically in a variety of canine sports. At 10 years of age, she began to show reluctance to enter the weave poles in agility. There were no obvious signs of lameness at that time, and the dog remained quite active. Following an impact to both hind limbs (hard landing on the teeter totter), a right hind lameness developed. Radiography was performed, and degenerative joint disease of the right hip was diagnosed. In addition, muscle atrophy was detected in the right crus and the thigh. Following rest and analgesic treatment, the dog’s overt lameness resolved, and she regained her former high level of activity. In the video taken three years later (while receiving chronic NSAID therapy), although she remained very active and enthusiastic, some difficulty is evident with certain movements, particularly backing up (diagonal position, curving path) and moving sideways (awkwardness).

### 2.3. House-soiling Problems

Chronic arthritic pain, especially in older animals [11] but also in overweight individuals, has been postulated as a cause of house-soiling in cats [14,31]. Several of the authors of the current article have observed individuals avoiding litter trays with high sides or going up or down stairs to access an area where the litter tray is kept, presumably due to the pain associated with accessing the latrine. These cases may respond with appropriate analgesia. A similar aversion to movement leading to house-soiling problems has also been reported in older dogs with chronic musculoskeletal pain [32].

A history of lower urinary tract disease has also been found to increase the likelihood of a house-soiling problem nearly four-fold [33] (odds calculated in [34]). More recently, concurrent health data from house-soiling cases [35] suggest that lower urinary tract disease may be particularly relevant to cats presenting with spraying behavior, with 12/18 (67%) of ongoing cases but only 3/18 (17%) of household matched control subjects having easily detectable changes; by contrast, the values for latrine related issues were more similar but still relatively high for both at 13/28 (46%) for ongoing cases and 10/28 (36%) for controls. House-soiling problems are less common in dogs but have been reported in association with urolithiasis [36]; this might have a similar pain association. Given the close association between interstitial cystitis and pain in humans [37], it seems reasonable to suppose that certainly some house soiling problems may be the result of either a current or learned association between painful micturition and the site of urination leading to avoidance of the litter tray. It has also been suggested that feline interstitial cystitis may cause more general anxiety in cats [38,39], a phenomenon that might also be pain mediated as has been found in people [37].

### 2.4. Attention Seeking Behavior and “Clinginess”

Comfort seeking, clinginess, and attention seeking are all widely recognized by owners as a response to pain in dogs [40], and thus it is not surprising that this behavior may become conditioned, especially in a species as sensitive to social reinforcement as the dog. While owner seeking behavior may be a well-recognized sign of disease and illness in dogs, it can present without overt signs of illness and thus appear to be a behavior problem. Attention seeking behavior takes many forms and is highly individualistic as owners may inadvertently reinforce behaviors of particular significance to themselves [41]. When sick, animals may learn what behaviors gain extra attention and resources, and this can develop into a more serious attention seeking problem, even after the illness has been successfully treated. The rate of these behaviors may decrease markedly (if not almost entirely) with analgesia without the need for specific behavior modification exercises when ongoing chronic (often musculoskeletal) pain is effectively managed. One such subject observed by an author had an unrecognized abnormal gait, especially after travel/exercise, and although no lesion could be identified, the problem was responsive to trial analgesia.

### 2.5. Star Gazing, Fly Snapping, and Other “Compulsive Type Behaviors

A potentially under-recognized manifestation of gastro-intestinal pain in dogs is star gazing behavior. This is described as an upward raising of the head and neck extension followed by staring at the ceiling or sky. Poirier-Guay et al., [42] report that a dog presenting with multiple consecutive episodes of star gazing daily was diagnosed with erosive gastritis with reflux esophagitis and that one week after implementation of treatment for these conditions, the star gazing resolved. The authors hypothesized that the star gazing was a manifestation of pain associated with the medical conditions [42]. Several authors [42,43] have suggested this behavioral manifestation may be similar to Sandifer syndrome [44] which is documented in infants with gastroesophageal reflux who show abnormal movements of the head, neck and trunk.

Fly snapping, also known as fly biting, air biting or jaw snapping, is a syndrome in which dogs appear to watch or see something not visible to humans and then snap at it. Some dogs will extend their neck and raise their head prior to fly snapping. In a study of seven dogs presented with fly biting [43], an underlying medical condition was found in all of them. They included gastric and/or duodenal eosinophilic or lymphoplasmacytic infiltration, delayed gastric emptying, gastroesophageal reflux, and Chiari malformation. When treated, 86% of them improved, with 57% showing complete resolution of the fly biting within a month.

Excessive licking of surfaces has been described as licking of any surfaces in intensity, frequency, or duration that cannot be justified by normal exploration of the environment. In some dogs, this behavior has been associated with gastrointestinal disorders such as eosinophilic and/or lymphoplasmacytic gastritis and/or enteritis, delayed gastric emptying, irritable bowel syndrome, chronic pancreatitis, gastric foreign body and giardiasis. When treated, 59% of dogs improved greatly in 3 months. When follow-up was prolonged to 6 months, the improvement went up to 76% of dogs. Interestingly, even if some dogs did not have a gastrointestinal disorder identified, the excessive licking of surfaces decreased following a course of hypoallergenic diet, antacid, and anti-emetic, suggesting nausea or possibly the discomfort/pain associated with hyperacidity could be a potential cause [45].

### 2.6. Pica

- Case study: Pica in a Labrador Retriever with hip dysplasia adapted from [46].

A 5.5-year-old female neutered Labrador Retriever presented with a 4.5 year history of pica (ingesting stones) requiring five laparotomies, three of which occurred in the previous year. Repeated laboratory tests (serum biochemistry, hematology, electrolytes) were unremarkable, and there were no other behavioral problems. Stone eating could occur in the presence or the absence of her owners, and on the most recent incident, the dog suddenly stopped whilst running on a pebble beach to ingest stones, previously having ignored them. On physical examination, the dog had a stiff hind limb gait and mild lumbar and bilateral hip discomfort. The dog’s owners were given advice on muzzle training and avoiding areas with stones until further medical tests could be conducted. Blood (canine pancreatic lipase, trypsin-like immunoreactivity/folate/cobalamin) and fecal (parasitology, including Giardia, culture, and sensitivity) tests were all unremarkable. Soon after, the dog required a further laparotomy to remove a stone, and at the same time, full thickness gastric biopsies were taken. Sparse *Helicobacter pylori* of unknown significance were identified with mild superficial chronic gastritis. Radiographs revealed mild bilateral hip dysplasia. Treatment was initiated with amoxicillin-clavulanate, metronidazole, and ranitidine and continued for 12 weeks until subsequent gastroscopic biopsies revealed minimal spirochetes; however, this did not alter the dog’s attempts to pick up stones. A 6 week analgesic trial (robenacoxib) for hip pain commenced. Within that time, the dog was reported to occasionally pick up a stone but would not swallow it and was also noted to be more active and playful. After a further 2 months of treatment, the owners reported the dog was no longer interested in stones. In this case, whilst gastrointestinal problems were considered, musculoskeletal pain appeared to be the cause of the pica.

Repetitive (apparently compulsive type) behavior, e.g., licking/chewing of the carpus [47] or tail, might also arise from pain [48], potentially leading to self-mutilation [49], presumably as the animal’s attention is drawn to the area of discomfort. Indeed, Denerolle and colleagues [47] highlight that acral lick dermatitis (“lick granuloma”) is often considered to be a behavioral problem but go on to describe several cases of other diseases causing lesions on the distal legs, which can mimic acral lick dermatitis, including lymphoma, an orthopedic pin, deep pyoderma, mast cell tumor, leishmaniasis, and (presumptive) sporotrichosis. Self-reinforcement may be obtained through repetition of the behavior [50], leading to a need for management of both pain and the compulsive element of the problem.

### 2.7. Other Examples from International Veterinary Behavior Meeting (IVBM) 2019

Many other forms of behavior problem can be caused by pain, and undoubtedly many cases remain undiagnosed due to a failure to recognize the relationship between clinical signs and pain. A failure to recognize the wider relationships between behavior and pain (considered in the following sections) is perhaps an even more common problem, but before discussing these, we conclude this section with a list of unpublished observations by the authors and the participants of a roundtable discussion of the topic at the 12th International Veterinary Behavior Meeting in 2019. There was a consensus on the validity of case studies relating to the following behavioral complaints being potentially pain mediated:

- Destructiveness when left alone (see also next session on secondary signs for a case study);
- Fear/anxiety for no apparent reason. A case observed by one author also engaged in trance-like states associated with an arched back (but without dorsal turning of the head). Stifle crepitus was apparent, and again the problem resolved with analgesic intervention, suggesting the episodes might be related to acute pain-induced spasms;
- Resource guarding, including protectiveness of the water bowl, has been widely observed by the authors in association with both musculoskeletal pain (e.g., hip dysplasia) and chronic gastro-intestinal disease. This sometimes appears in a mild form as a tolerated issue secondary to other more overt forms of human directed aggression. Spontaneous resolution of the behavior has been observed when the animal was given analgesia for putative musculoskeletal pain identified as part of the wider medical evaluation of the case;
- Aggression to the owner as a result of anal gland impaction in cats and dogs;
- Refusing to go for a walk, freezing on a walk, or refusal to enter a part of the house with potentially slippery flooring (see also the case in the next section);
- Disturbing/waking the owner at night—this might also be a sign of age-related brain degeneration in cats, and it is important to recognize when it is pain-related, since treatment is often very successful in these cases.

## 3. Unidentified Pain Underpinning Secondary Concerns within the Initial Behavior Problem

### Incompletely Managed Cases

In some cases, a primary complaint may be behavioral, but one or more of the signs may be related to pain. This is perhaps a less commonly recognized situation but no less important, as failure to recognize this can result in some aspects of the problem improving with a recognized behavior modification plan but the other aspects associated with pain appearing refractory to treatment. Indeed, from a diagnostic perspective, this feature of a case may be an indication to investigate this type of issue. If the pain aspect is not managed, not only does the patient suffer, but also the owners may be left frustrated at having got “so far but no further” with their management of the case, and indeed the case might even relapse as a result. These types of case are not well documented in the literature, but we describe a couple of illustrative case studies below. In the first case, the pain related sign may appear to be an integral part of the presenting syndrome (separation related problem), whereas in the second, the pain related issue seems initially to be a secondary issue of concern. In the third case, we highlight how a painful lesion may result in a behavior patient presenting as a potential relapse.

- Case study: Destructiveness related to pain within the complex of signs presented as a noise fear induced separation related problem

A 9-year-old male neutered Border Collie who had been in the owner’s home since 14 months of age presented with a 7 year history of being scared of jet planes as well as generalized anxiety that was exacerbated when he was left alone, and whilst many of the separation related signs could be controlled, there was a persistent problem related to destruction when left alone. The dog would dig through carpet and damage door frames, and thus the owner had taken up carpets in some parts of the home, kept him in a tiled room, and also tried to confine him to a crate when left alone. They found that neither giving the dog free access to the house nor crating made any difference to the frequency of destructiveness behavior. The case was referred by a veterinarian with an interest in behavior, because they were not making the expected progress. Treatments might be helpful for a short time, but the dog would soon relapse. The medication history for the problem included 9 months on selegiline, 1 month on propranolol and phenobarbitone, 2 years on clomipramine initially with alpha casozepine but subsequently replaced with alprazolam for 4 months, 2 months on paroxetine with a further 2 months on this medication combined with oxazepam. In the 3 months leading up to consultation, trazodone had been used in place of oxazepam, but it was agreed to start weaning the dog off all meds before the behavior clinic visit. Video assessment of the dog’s behavior showed that he would engage in relatively fixed, “compulsive-like”, short bouts of scratching behavior with one or the other forepaw after lying down. The dog had a varied history of suspected hindlimb injuries, including a cruciate tear, but of particular note was an injury to the left hock when the dog was about 2 years old. This was radiographed at the time, and since no bony damage was apparent, the condition was treated conservatively with non-steroidal anti-inflammatory drugs and rest. Upon clinical examination, the Achilles tendon appeared thickened but was not painful to touch. Initial behavioral advice consisted of continuing to wean the dog off all previous medications, with a view to introducing a combination of phenobarbitone and propranolol again, not only because this combination has been recommended for anxiety and especially noise fears with a strong autonomic component [51], but also in case the digging behavior was seizure-related. The owner was advised to continue enhanced previous management recommendations at home and to provide the dog with a digging box (child’s sandpit with turf) with training aimed at encouraging redirection of the behavior towards it. The owner was also provided with a diary containing the identified eliciting contexts with which to monitor the behavior (as per King et al. [52]) and encouraged to make regular video recordings when the dog was alone. After six months, the dog was generally doing well; he seemed more settled and less anxious, but the digging had not resolved. A radiograph of the left hock of the patient was then requested. This revealed extensive ossification of the Achilles tendon and spurs on the calcaneus. A course of meloxicam was therefore recommended with all other management remaining the same. After 1 month, the owner reported that the patient seemed more relaxed at home, and no digging was observed on any of the videos (several recordings had been made weekly, and some of these were 4+ h long). In humans, plantar fasciitis is a painful condition of the calcaneus characterized by stabbing pain when an individual starts an activity involving the foot [53], and we suspect a similar condition was affecting this dog. Although it is normally a self-limiting condition in people, in this case, there was a clear pathology to explain its persistence. We suspect the foreleg digging was a form of redirected activity associated with discomfort following rest. Whilst the destructiveness was not the primary complaint of the owner, its resolution was critical to the owner’s perception of the complaint and also the dog’s well-being; indeed, we suspect that previous failure to resolve the pain may also account for the relapse given the relationship between anxiety and chronic pain [54].

- Case study: Apparently secondary problem with walking related to pain in a dog with resource guarding

A 1-year-old male neutered Cockapoo belonging to first-time owners since he was 8 weeks old was presented for aggressive behavior over items. The dog was friendly with people and dogs when items were not involved. The dog had a history of being trained using reward focused methods, and the owners commented how he also did not seem to like to go for walks. The resource guarding behavior was successfully managed using standard behavior modification, but the reluctance to go for a walk did not improve. Further investigation of this issue revealed that the dog would back off and move away when the owner approached with the walking harness; he also appeared to be more generally avoidant of handling (the owners had come to the conclusion he was not very sociable, which they found disappointing). Despite this, the owners were still trying to walk the dog twice a day, but the dog was now stopping on walks (sitting down close to the home) on a variable basis; the problem was getting worse and more frequent in recent months. Sometimes he could be persuaded to walk using a food lure but not always; he would also now run away from the walking harness and snap, but there seemed to be less of a problem if they used a flat collar (the owners had previously been advised when he was a puppy that harnesses were better for dogs and thus had not wanted to change this). In the clinic, when he was taken out for a brief walk, his gait appeared stilted. The case was referred for further diagnostic work up, and in the interim, the owners were encouraged to use the collar for walking, to keep the walks short (15 min), and not to force him to walk but to make them up-beat. Upon veterinary examination, the veterinarian could find nothing on physical examination, but radiographs revealed marked bilateral hip dysplasia with joint laxity evident when the dog was anesthetized. The dog was then prescribed NSAIDs for 4–6 weeks. This enabled the owners to extend the walks to about 20 min each, and they used the garden more for exercise; however, there were still significant issues of aggression. Paracetamol was then added to the treatment, and some improvement in the target behaviors was seen, and the owner also noticed that the dog had begun to stretch his back legs occasionally. Gabapentin was then added, and a further marked improvement in the risk of aggressive incidents was evident alongside a further reduction in stopping on walks, although he was still occasionally avoidant. Over the course of the next few weeks, the owners continued to improve the management of the dog and were able to use the harness for walks.

In this case, the primary concern for the owner was their relationship with their dog, which was not what they thought it would be as novice owners. This was epitomized by the dog’s resource guarding behavior, which was the focus of their initial complaint. Although this resolved with a standard behavior modification program, the lack of enjoyment when trying to walk the dog meant the initial problem was not fully resolved due to the secondary issues being tolerated. Only once the first issue had been addressed did the significance of the secondary walking issue become apparent. The avoidance of the harness did not respond well to a desensitization and counterconditioning program until pain medication was used, and it took some time to find the combination for this particular subject. In our experience, this is not an uncommon finding, i.e., several combinations may be required, and the involvement of pain cannot be ruled out following a single negative result. The radiographic changes and the positive Ortolani test obviously helped to provide evidence for the general practitioner that the potential for pain needed to be pursued, but, in our experience, many cases may lack such evidence. Video footage can be invaluable, and it seems many dogs may inhibit or mask their behavior while stressed and thus show few signs in a short general practice veterinary appointment; by contrast, over the course of a much longer behavior consultation, more signs may be evident. For the general practitioner, one way around this problem is to ask the owner to video the dog at home so that the gait can be carefully evaluated.

- Case study: Pain as a cause of apparent relapse of a managed case

A 4-year-old, 25 kg male neutered mixed breed dog was originally presented for attacking one of the other dogs in the house. Additional issues included aggression towards unfamiliar dogs, unfamiliar people, and general high arousal and hypervigilance. The patient was treated successfully for the interdog aggression and generalized anxiety with fluoxetine, management changes, and behavior modification protocols. The patient had been behaviorally stable for 6+ months until approximately a year later, when he presented with an apparent reoccurrence of his anxiety. No changes in household dynamics, medication, management, or behavior modification by the owner were noted. However, the owner now observed an increase in the frequency of the dog licking his lips, pupil dilation, heightened sensitivity to environmental noises (indoors and outdoors), and hypervigilance. The patient had not had a physical examination for about 10 months. Mild periodontal disease was reported at this time but no other findings, and the patient’s chart had a warning that he will bite if handled anywhere near his hind end. During the consultation, the patient was observed to be off-loading weight from the left rear limb while standing and to have a stilted/stiff hind limb gait. A 4 week NSAID (grapiprant) trial was recommended with all other treatments continued as previously prescribed. Four months later, the owner reported a decrease in hypervigilance and noise sensitivity, decreased intensity of responses to people and dogs on walks, and increased playfulness since starting NSAID treatment.

As illustrated by the case studies above, the importance of pain related behavioral changes may only become apparent after behavior modification for the primary complaint. Points to note in this regard are slow or lack of progress on certain signs within the complaint and frequent unexplained relapsing of certain signs. In some cases, the behavioral issue may be secondary to a medical one, and the owner may not be seeking help but is pleased when they see the behavioral problem resolve alongside the medical issue. An example of this from the IVBM was an account of a cat being treated for small cell lymphoma, whose owner commented how its pica disappeared as its medical treatment progressed. Another account related to a female spayed dachshund being treated for separation related problems alongside neck/back pain, whose nosing of the owner disappeared when the pain was managed. This might have been a form of care soliciting behavior associated with the discomfort. Perhaps the most common secondary sign that appears to resolve with the treatment of pain relates to various behaviors that, prior to the use of analgesia, were being interpreted by the owner as some form of stubbornness.

Dogs may show signs of pain when walking on very cold (e.g., ice or snow) surfaces, especially in countries with regular temperatures below −10 °C. These signs include: lifting or shaking a paw, unwillingness to walk (to move any limb), crouching or collapsing (all limbs flexed and the ventrum in contact with the ground), and vocalization (crying or whimpering). Central sensitization in relation to pain [55,56] may be associated with hypersensitivity to cold [57,58] or heat [59] as well as to pressure (allodynia or hyperalgesia) [60], and we speculate that this could be a sign of painful conditions (e.g., degenerative joint disease) in pet dogs; it may also be a performance-limiting factor in service dogs living in climates with temperature extremes (e.g., cold winters). A recent study showed somatosensory sensitivity to touch, heat, and cold in dogs affected with hip or stifle osteoarthritis at the affected joint as well as on the cranial tibial muscle and the dorsal metatarsus [55]. Although the plantar surface of the foot was not tested, it seems reasonable to suggest, given our anecdotal observations, that hypersensitivity could extend to this region.

We have also seen numerous cases where the owners report what they think is a bizarre behavior related to the animal avoiding certain rooms in the house for no apparent reason; upon closer enquiry, it has become apparent that the nature of the flooring may be the source of this behavior (which is not the primary complaint). We hypothesize that the rooms may be avoided because the animal has difficulty coordinating its movement on certain types of surfaces that are slippery, leading to avoidance when the animal has pain, especially in the hips or the shoulders. A case study including this feature is given in the next section on the exacerbation of signs due to pain.

## 4. Exacerbation of One or More Signs of Problem Behavior as a Result of Pain

### The Influence of Mood Changes Associated with Pain on Problem Behavior

Sometimes pain is not the cause of the problem but may exacerbate it. Conditions such as pain induce a negative cognitive bias [61,62], which can be expected to exacerbate a wide range of problems associated with negative affective state, such as anxieties, fears, and frustrations. However, this relationship is probably bidirectional [63], with animals suffering from problems relating to the latter affective states also potentially more sensitive to pain. As already mentioned, if we operate in accordance with the precautionary principle, then whenever there is a suspicion of pain involvement in a behavior case, treatment should consider the management of both pain and other negative affective states from the outset. This applies even if no overtly painful lesion can be found if we wish to safeguard the well-being of the patient. Obviously, if we start this sort of multimodal treatment regime, it might be difficult to establish how much each is contributing to the problem unless some aspect of management is changed; this can be done once the problem is under control with the clinician making the judgement as to which aspect of the treatment program can be eased first. However, in some cases, this might also arise unintentionally, as in the following case, where modification of the pain management regime resulted in an exacerbation of signs.

- Case study: Exacerbation of aggression following unscheduled reduction of pain medication

A 2-year-old male neutered black Labrador had presented with a history of growling at the owners (more to the husband than the wife) since it was 6 months old. The dog also initially avoided walking across the kitchen floor to go outside, and this avoidance behavior had spread to any surface in the house without carpeting. Growling would often occur when one owner walked by while the dog was resting, and if asked to go out at this time, he could turn and bite. The dog had a potential history of injuring himself prior to the problem, since he was seen limping, but the owner had no idea what he had done. The referring veterinarian could not find any reason for the patient to be limping and had referred the case to a veterinary orthopedist. Multiple radiographs had been taken, but no abnormalities were noted. After 2–3 days of treatment with carprofen, he was no longer limping, and treatment stopped. During the behavior consultation (approximately 18 months later), no obvious areas of pain were evident on palpation. However, both of the dog’s stifles were rotated out when he was standing. He tended to avoid weight bearing on his left hindleg. Further radiographic examination of his hips and knees was recommended alongside gabapentin for suspected pain and trazodone for his anxiety. Behavior management focused initially on reducing the risk and the avoidance of triggers of the aggressive behavior. The owners were advised not to force the dog to go outside, to put carpet runners down to help him be more comfortable walking through the kitchen to go outside, and to toss treats towards him as they walked past him when he was resting. The radiographs showed no significant findings. Six months later, the owner reported the dog was no longer growling at the female owner, but there was still growling towards the male owner, who was ignoring the advice not to initiate contact with the dog when he was lying down. The dog appeared to have a short stride length with crossing over of hind limbs accompanied by inward rotation of the right hind foot. His rump would also sway slightly as he walked. He would sit with his legs tucked under his abdomen and pointing cranially. There also appeared to be atrophy of the semimembranous evident on the left hind limb. Since he still appeared anxious, the dose of his medication was increased (trazodone 75 mg–100 mg twice a day, gabapentin 300 mg BID) and supplemented with paroxetine (45 mg once a day. In addition, aspects of the behavior modification plan (centered on him trading low value items for higher ones) were reinforced with reassessment in 3 weeks. Within 3 months, the owners had noticed a marked improvement (described by them as “at least 95% better than he was”), and they were much more confident around him. About 8 months later, the owners reported that the dog had started growling again at them, the husband’s friends, and their other dog, with whom the patient had always played well. The owner noticed that the dog struggled to get back up after lying down for a while. Though he did not appear to limp when walking, sometimes his back legs shook when he was standing. A physical examination of the patient was not possible at the time of this follow up consultation due to his growling; however, it became apparent the owner had reduced the gabapentin dose because she was running low. This was restored to the recommended dose with the suggestion that an NSAID trial may be useful. Six months later, the patient was doing well again, and the problem seemed to be managed well for some time after this as long as the pain continued to be managed. This included the use of carprofen 75 mg BID. Despite further radiographic assessment of the spine and the hind limbs, no physical lesion could be identified. In the authors’ experience, this is not an uncommon finding and can deter some veterinarians from implementing an effective pain management regime. However, we hope that the wider recognition of the potential role of pain in problem behavior through case studies and articles such as this will encourage more proactive management of these cases.

It is important to consider pain in any behavior case where some aspect of the severity of the problem seems to be out of proportion to the cause. This might be the intensity of the signs, e.g., readiness of the dog to run away as fast as possible when scared, the number of signs shown, or the extent to which the problem generalizes to other stimuli or the wider context. The latter is noted by Barcelos et al. [2] in relation to dogs showing aggressive behavior, with those in chronic pain tending to show aggressive behavior towards a wider range of subjects (familiar and/or unfamiliar, humans and/or dogs). Fagundes and colleagues [3], in a qualitative analysis of noise sensitive dogs with and without musculoskeletal pain, identified several factors relating to the nature of the response that might be suggestive of pain involvement. In particular, there appeared to be a greater tendency to avoid a very wide range of locations associated with the noise in these animals compared to non-pain controls. For example, a dog in pain who experiences a sudden loud noise when out on a walk may no longer get out of the car if it is parked in the same place as before, even though it may be several miles from the initial incident. We suspect that, when these animals tense (in response to the noise), they experience greater pain, and this may explain the wider generalization of the response. Dogs in pain would also tend to hide when in pain rather than seek the owner, and this might be because contact with the owner may be associated with hugging or cuddling in an attempt to reassure the dog, but this action may exacerbate the pain. Fagundes et al. [3] also found that dogs in pain were more likely to develop the problem later in life (average age of onset of noise reactivity in pain related cases was 6 years of age compared to 2 years of age in pain-free dogs). In addition, although 6/10 of the pain-free, noise reactive dogs responded to all loud noises, all (10/10) of the painful noise reactive dogs responded to all loud noises. However, pain does not necessarily affect all aspects of the problem equally, and the following case history, in which the anxiety was quantified by the owner before and after analgesic medication, is a good illustration of this:

- Case study: Exacerbation of certain forms of avoidance in a dog with chronic pain

**Figure 1:**
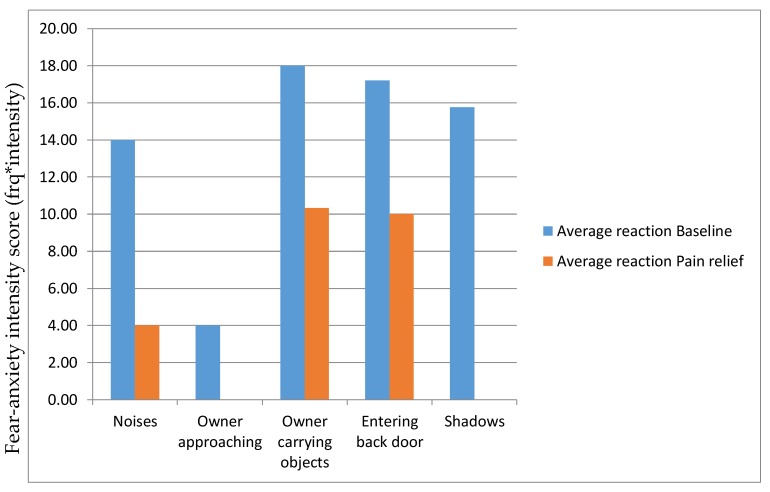
Intensity of fear/anxiety in five contexts for a patient scored before and after receiving analgesia. Frequency score: never = 0, rarely = 1, frequently = 2, every time = 3; intensity score: 1 = small amount − 5 = extensive amount.

A 3.5-year-old male neutered 21 kg crossbred dog was referred for evaluation of its anxiety. Fear/anxiety was shown in relation to noise, the approach of the owner, the presence of the owner carrying objects, when entering the back door, and apparently towards certain shadows. Following the initial consultation, it was noted that a lower canine was impinging on his hard palate, and it was recommended that this be extracted. In addition, it was noted that the dog appeared to have hip dysplasia and an angular deformity of both forelimbs. The dog was therefore started on a course of meloxicam once a day after the tooth had been extracted and the mouth lesion had healed. After about 4 weeks, the dog’s anxiety in the contexts that had been identified previously was re-evaluated using the scale described in McPeake and Mills [64]. The dog’s fear response to the approach of the owner and shadows had disappeared, and the response to the other stimuli was reduced to a varying extent. Quantified results are shown below (Figure 1).

At this point, it is also worth drawing attention to the similarity between signs of chronic pain and cognitive dysfunction in older animals, which has been highlighted in the popular veterinary press [65]. Many older dogs may suffer from chronic painful conditions as well as a degree of cognitive decline [66], including pathological processes such as cognitive dysfunction [67]. Chronic pain may not only appear symptomatically similar [65], but it may also exacerbate both normal aging and cognitive dysfunction. It should be considered that, rather than an animal having either pain or one of these conditions, it may have both. A partial response to treatment of one condition could indicate a degree of refractoriness or may indicate that treatment of both pain and cognitive decline is required. It is clear that not only veterinarians but also owners need to become much more aware of the potential to help older animals [67].

## 5. Adjunctive Behavioral Signs Associated with Pain

Most veterinarians will be familiar with signs of pain such as shifting between limbs when standing, an abnormal gait, propping oneself up against convenient objects, interruptions to gait/and sudden freezing, an unusual approach to lying and sitting, an unconventional sitting or lying posture, unusual/hesitant defecation and urination behavior, and nibbling or scratching of a specific area, especially if sudden and accompanied by a skin flinch or yelp. In the clinic, restlessness, including excessive sniffing, as well as signs of anxiety and owner comfort seeking behavior may also be indicative of chronic pain. A growing concern is that some of the behaviors are so common in a particular type/breed of dog that they may be becoming normalized, and their clinical significance is going unrecognized as a result. Recently, Rohdin and colleagues [68] reported that more than three quarters of pugs with an abnormal sitting posture (one leg tucked under the body, sometimes referred to as “lazy sit”) also had an abnormal gait. Dogs with an abnormal gait were not only more likely to be irritable, reluctant to go for a walk, and unable to jump up, but they also had a much higher prevalence of adjunctive behaviors, which might be less widely associated with pain. These included abnormal scratching of the head and the neck, air licking, and fly snapping. More recently, Rusbridge and colleagues [69] noted that syringomyelia in Cavalier King Charles spaniels also appeared to be associated with “phantom scratching” (as well as avoidance of touch, which might easily be presented as owner avoidance or petting aggression, etc.), whereas otitis media with an effusion was not associated with head scratching/rubbing. Thus, these signs may indicate deeper central nervous system pathology rather than ear disease. Whether these behaviors are actually signs of pain or are associated with it in other ways remains uncertain, and it is for this reason we refer to them as adjunctive behaviors associated with pain rather than signs of pain. They are nonetheless clinically valuable for both behaviorists and general veterinary practitioners as an indication for closer examination and consideration of a pain focus. Other adjunctive behaviors of importance recognized by a range of clinicians (affirmed in the discussion at IVBM 2019) include yawning, frequent body stretching, body shaking with or without initial scratching (and interrupted body shaking), and excessive licking of others.

- Case study: Normalization of pathological changes

An 8-year-old male neutered Terrier Cross was presented with a sudden onset of destructive behavior when left alone within the preceding two weeks. The owners would often return home to find ornaments knocked off the window ledges/tables and chunks of wood bitten off furniture. The windowsill could be covered in saliva and the curtains pulled down. The dog had been to see another behaviorist, and a program for desensitization to the leaving routine was recommended. At consultation, the owner reported some other changes in behavior a few weeks prior to the onset of destructiveness when left alone. This included a decrease in play behavior, appearing “depressed” (i.e., walking with head and tail down), worsening of noise sensitivity, and appearing “anxious” some evenings. The latter consisted of a variable sudden onset panting, shaking, and trying to squeeze into small corners while the owners were present. The dog had a wire cruciate repair to the left hind limb just over two years previously. In the clinic, he was observed to have a stiff hind limb gait with reduced weight bearing on his left hind.

Repeat radiographic reevaluation of the stifles and the hips was recommended. A longer trial of analgesia (4–6 weeks) was also recommended while recording destructive behavior in a diary. Some basic management advice (keeping breakable items out of reach) and a simple exercise in creation of a safe haven was also given in the interim. The referring veterinarian notes indicated the following: that he thought the stifle and the patella were stable; that the left stifle metal work was palpable but, as there were no swellings/pain nor lucency around the pins, he did not think the implants were causing a problem. Some mild periarticular osteophytes were noted on the stifle compared to the right, with mild degenerative joint disease that was consistent with the previous fracture. On this basis, he had decided to tell the owner that, in his opinion, the changes shown would not cause behavioral problems in the average dog. Nevertheless, the veterinarian continued with the recommendation of a longer duration of analgesia for 6 weeks. Within 2 weeks of initiating treatment with meloxicam, the destructiveness upon separation had completely resolved, and the owners had noticed an increase in play behavior and cessation of “anxiety” and “depression”-like signs within 2 weeks of starting analgesia. Due to the positive response to analgesia, it was continued past the 6 week period. The improvements were maintained 3 months later, after which, follow up was ended.

These sorts of comments are not uncommon and not only reflect a failure of some veterinarians to appreciate the individuality of pain responses in animals but can also seriously undermine important treatment aimed at safeguarding the patient’s well-being.

## 6. Conclusions

At this stage, we can only speculate about the potential adaptive mechanisms underpinning some of the observations discussed above. A number of explanations are possible. Animals in pain or who experience any illness may feel more at risk and thus express higher levels of anxiety. This may carry with it the potential for frustration, as they are unable to access the resources they want or perceive certain resources as more valuable, since the cost of access increases with the degree of pain associated with gaining it. Some displacement behaviors (including some of the adjunctive behaviors described here that are sometimes referred to as “calming signals”, such as yawning [70]) may serve to increase endorphin release and thus alleviate some of the discomfort, as might engaging in a social interaction with an owner. The latter could prompt attention seeking and distress when an owner is not available.

Where pain is suspected or potentially implicated from a theoretical perspective in a patient referred with a behavior problem, it is important to keep an open mind about the potential influence that it may have on that individual. We should not discount the potential role pain plays in such cases, even if the link appears unlikely or is not yet documented in the published literature. Trial analgesia may not be sufficient to definitively confirm a pain focus, but, in any case, it will safeguard the welfare of the patient, demonstrate compassion for the patient, and may negate the need for a behavior modification program that the owner may otherwise struggle to undertake. For these reasons, it is our recommendation that it is better to treat suspected pain first rather than consider its significance only when the animal does not respond to behavior therapy.

In order to increase awareness of this issue, there is undoubtedly a need for all encountering these cases to document them in the form of case reports or case series. Case reports do not provide definitive evidence, but they are valuable for highlighting important observations for the profession. We referenced several here that report specific accounts of pain-related behavior problems (e.g., 8, 10, 49) and supplemented them with additional reports to illustrate key points within the article in the hope that this will encourage greater publication on this important topic. Case series do not have to be extensive but potentially enable us to discern specific features for identifying these sorts of behavior problems that might be suggestive of pain. These not only increase awareness of the involvement of pain in problem behavior but also can help clinicians learn how to better differentiate these cases from medically normal ones presenting with the same complaint (see: [2] for aggressive behavior and [3] for noise fears).

## Figures and Tables

**Table 1:** Prevalence of pain-related behavior problems in dogs seen in various referral clinics of the authors.

Location	Bristol	Lincoln	Pennsylvania A	Pennsylvania B	Missouri/Illinois	Barcelona
Dates	2019	2018–2019	2017–2018	2018–2019	2018–2019	2018–2019
Proportion (%) of behavior cases where a painful condition was suspected	68%	82%	23%	29%	79%	28%
Of these, the most common sources of pain seen	1. Hip—71% 2. Stifle—54% 3. Carpus/tarsus—29% 4. Spine—14% 5. Abdominal (unrelated to a specific disease)—3% 6. Possible allodynia—3%	1. Hip—63% 2. Stifle—24% 3. Spine—39% 4. Shoulder—1% 5. Elbow—15% 6. Carpus/tarsus—1% 7. Gastro-intestinal (GI)—1% 8. Dental—1%	1. Hip—17% 2. Spine—23% 3. Elbow—4% 4. Carpus/tarsus—4% 5. Ear—13% 6. Stifle-26% 7. Dermatologic lesions—13%	1. Hip—31% 2. Spine—31% 3. Stifle—21% 4. Ear—14% 5. Carpus/tarsus—3%	1. Hip—44% 2. Stifle—42% 3. Spine—32% 4. Gastro-intestinal—27% 5. Other dermatological 27% 6. Ears—26% 7. Elbow—8% 8. Other musculoskeletal—4%	1. Hip—8% 2. Stifle-knee—1% 3. Carpus-tarsus—3% 4. Shoulder—2% 5. Neck—3% 6. Spine—7% 7. GI—2% 8. Ear—1%
Proportion (%) of these cases with some form of confirmation of diagnosis	79%	56%	61%	79%	72%	16%
Methods used for confirmation beyond observation of clinic behavior	Physical exam—54% Response to analgesia—48% Diagnostic imaging—23%	Diagnostic imaging—66% Response to analgesia—24% Physical exam—9% Referral to specialist for GI workup—1%	Physical exam—86% Response to analgesia—8% Response to wound management—8%	Physical exam—77% Diagnostic imaging—14% Response to analgesia—9%	Physical exam—70% Response to medical treatment other than analgesia—55% Response to analgesia—52% Diagnostic imaging 35% Referral to specialist 18%	Physical Exam—64% Diagnostic imaging—43%
Proportion (%) of cases for which there were other medical conditions of concern (all or some of which might also be painful conditions)	23%	15%	25%	15%	45%	32%
Of these, the most common concerns related to:	Gastrointestinal—8% Neurological—2% Glaucoma—2% Anal sac disease—2% Dermatological—9%	Gastrointestinal—7% Neurological—3% Hypothyroid—2% Dermatological—2% High blood pressure—1%	Gastrointestinal—8% Dermatological—11 Neurological—4% Respiratory—2%	Gastrointestinal—7% Dermatological—6% Respiratory—1% Neurological—1%	Dermatological—13% Gastrointestinal—7% Autoimmune—4% Endocrine—4% Dental—3% Neurological—2%	Gastrointestinal—9% Neurological—10% Dermatological—4% Hormonal—5% Ophthalmic—3% Liver—1% Cancer—2% Oral—2% Respiratory—1%

## Supplementary Materials

The following are available online at https://www.mdpi.com/2076-2615/10/2/318/s1, Video S1: Video of a Weimaraner performing canine freestyle routine.
